# Anemia

**Published:** 1911-06

**Authors:** 


**Anemia.** By **Dr. P. Erlich**, Frankfort-on-Main, and **Dr. A. Lazarus** Berlin. Part I, Vol. 1, Normal and Pathological Histology of the Blood. Second German edition. Translated by **H. W. Armit,** M.R.C.S., L.R.C.P., London. Octavo pp. 230. Illustrated New York: Rebman Co. (Cloth, $4.00.)

This work, Part I of Volume I, considers the normal and pathological histology of the blood. Coming as it does from one of the pioneers and great masters in the domain of hematology, it deserves careful reading and study. The original German is the second edition and appears ten years after the first edition. It has naturally been enlarged and contains all the recent advances in the study of the blood. Obviously, the book is largely rewritten.

Professor Erlich, himself, busy as he has been during the past decade with his researches in immunity—a field in which he has attained as great a success as in that of hematology—has been unable to personally revise his original work, but it can really be considered his own as it was planned by him and rewritten by his former assistant, Professor Lazarus of Berlin, and his former co-worker, Dr. Nargeli of Zurich, both of whom were trained in Professor Erlich's laboratory, the Royal Institute for Experimental Therapeutics at Frankfort.

Chapter I. concerns itself with the methods of the examination of blood and gives the essential facts requisite for its thorough study—the less important minutiæ being omitted. Chapter II. takes up the morphology of the blood particularly the red corpuscle, normal and pathologic. Both chapters comprise about 75 pages of interesting reading and are from the pen of Professor Lazarus.

Chapters III. and IV. are written by Dr. Nargeli and treat of the leukocyte and blood platelet. The varieties of the leukocyte, both normal and pathologic, are fully described—the origin, methods of demonstration and clinical significance of each type thoroughly considered.

It should be noted that controversial as any theoretical question usually is, and that of the blood has been no exception, the theories of Erlich although not universally accepted even today are none the less adhered to by a larger number of authorities from year to year. Even with the more refined methods of study and research very little has been changed in the ideas advanced by Erlich and his school.

Credit is due to Dr. Armit for his admirable rendition of this master work into English. The original is now more readily accessible to the English and American practitioners, particularly to those who are not able to read scientific German with any degree of fluency.

The publishers deserve commendation for bringing out the book and for the excellent plates which fully illustrate the cytology of the blood. It is indeed a characteristic of the Rebman house to make their illustrations well nigh perfect. L. K.

